# Infantile hemangiomas: risk factors for complications, recurrence and unaesthetic sequelae^☆^

**DOI:** 10.1016/j.abd.2021.05.009

**Published:** 2021-11-27

**Authors:** Letícia Gaertner Mariani, Lílian Moraes Ferreira, Diego Luiz Rovaris, Renan Rangel Bonamigo, Ana Elisa Kiszewski

**Affiliations:** a Postgraduate Program in Pathology, Universidade Federal de Ciências da Saúde de Porto Alegre, Porto Alegre, RS, Brazil; b Faculty of Medicine, Universidade Federal do Pampa, Uruguaiana, RS, Brazil; c Department of Physiology and Biophysics, Instituto de Ciências Biomédicas, Universidade de São Paulo, São Paulo, SP, Brazil; d Faculty of Medicine, Universidade Federal do Rio Grande do Sul, Porto Alegre, RS, Brazil; e Service of Dermatology, Santa Casa de Misericórdia de Porto Alegre, Universidade Federal de Ciências da Saúde de Porto Alegre, Porto Alegre, RS, Brazil; f Department of Internal Medicine, Universidade Federal de Ciências da Saúde de Porto Alegre, Porto Alegre, RS, Brazil

**Keywords:** Hemangioma, Propranolol, Recurrence, Risk factors, Scars

## Abstract

**Background:**

Infantile hemangiomas (IH) occur in approximately 4% to 10% of the pediatric population. The identification of clinical subtypes and conditions that indicate increased risk for complications is essential for therapeutic success.

**Objectives:**

To identify risk factors for complications, recurrence and unaesthetic sequelae.

**Methods:**

Retrospective cohort of patients with infantile hemangiomas undergoing follow-up at the Dermatology Service of Universidade Federal de Ciências da Saúde de Porto Alegre, between 2006 and 2018.

**Results:**

190 patients were included; 24% had some type of complication, ulceration being the most frequent, and 86% required treatment. On correlation, ulceration was statistically related to mixed IH (*p* = 0.004), segmental IH (*p* < 0.01) and location in the gluteal region (p = 0.001). The mean time of treatment with propranolol was 12.7 months. Patients with PHACES syndrome and segmental infantile hemangioma required longer treatment (p < 0.001 and p = 0.0407, respectively), as well as those who started treatment after five months of life (p < 0.0001). Recurrence occurred in 16.6% of the treated patients, all-female; 94% were located on the head and neck (mainly on the upper eyelid, cyrano, S3 segment, and with parotid involvement); 61% and 38.8% were of the mixed and deep subtypes, respectively. Approximately 1/3 of the patients had some unaesthetic sequelae.

**Study limitations:**

As this is a retrospective study, data and photos of some patients were lost.

**Conclusions:**

Mixed and segmental hemangiomas are risk factors for ulceration and sequelae. Recurrence occurs more often in females and segmental hemangiomas. Segmental infantile hemangioma and PHACES syndrome require a longer time of treatment. Specific protocols are required for infantile hemangiomas with a high risk of recurrence.

## Introduction

Infantile Hemangiomas (IH) are the most common benign vascular tumor of childhood, occurring in approximately 4% to 10% of this population.^1^, ^2^, ^3^, ^4^, ^5^, ^6^ They can differ considerably in size, growth, location, and depth. In most cases, they are small, sporadic, and uncomplicated.^7^, ^8^

Morphologically, IH are classified as superficial, deep, or mixed. Regarding their extension, they can be focal, segmental, or multifocal.^1^, ^2^, ^3^, ^6^ The focal subtype is the most common, while the segmental subtypes – which occupy a subunit of the body – are often associated with complications.^3^, ^9^, ^10^, ^11^ Patients with segmental lesions on the face or neck may present with PHACES syndrome, which represents a spectrum of associated abnormalities (posterior fossa brain malformation, hemangioma, arterial, cardiac, ocular, and sternal abnormalities).^10^ Cyrano hemangiomas are those located on the nasal tip.

IH occur more often in preterm, female, and low-birth-weight infants.^9^, ^10^ Other risk factors associated with a higher incidence of IH are: older maternal age, Caucasian ethnicity, multiple pregnancy, placenta previa, and preeclampsia.^3^ Although they are usually sporadic, a family history of IH increases the risk of hemangioma, suggesting that a genetic predisposition may be implicated in the pathogenesis of IH.^1^

Regarding the natural history, it is generally divided into phases: (1) occurrence at birth or soon after; (2) early proliferation; (3) late proliferation; (4) stabilization (plateau); (5) involution.^1^, ^2^, ^3^ Most IH are not present at birth but typically appear in the first weeks of life, while some patients have a precursor lesion at birth: a small red papule, telangiectasia, hypochromic macula (corresponding to an anemic nevus), or pseudo-ecchymoses. The tumors grow rapidly for six to ten months (proliferative phase), with most reaching their maximum size between three and five months of age and slowly regressing by seven to ten years (involution phase), with complete regression seen in 90% of cases at four years of age.^6^

Patients diagnosed with IH undergoing follow-up at a university hospital in southern Brazil were analyzed aiming to understand the epidemiological profile, clinical and therapeutic aspects of these patients, and to identify conditions associated with complications, recurrence, and unaesthetic sequelae.

## Methods

This is a retrospective cohort study of patients with IH treated at the Department of Pediatric Dermatology of Universidade Federal de Ciências da Saúde de Porto Alegre, Brazil between 2006 and 2018. This study is in accordance with the principles of the Declaration of Helsinki and was approved by the Research Ethics Committee under number 1973303. Free and informed consent was obtained in all cases.

The diagnosis of IH was a clinical one in most cases, except for deep IH, which required complementary exams such as Doppler ultrasound, computed tomography (CT), or nuclear magnetic resonance (NMR). For SACRAL (spinal dysraphism, anogenital, cutaneous, renal, and urologic anomalies, associated with an IH of lumbosacral location) syndrome, MRI of the spine and pelvis was performed in the diagnostic context; whereas patients with PHACES syndrome were submitted to Computed Tomography (CT) and/or head MRI. The diagnosis of PHACES syndrome was performed according to the diagnostic criteria published by Metry et al. in 2009.^12^ Patients with multifocal IH were submitted to total abdominal ultrasound to rule out liver involvement.

The unaesthetic sequelae included anetoderma, redundant skin, fibroadipose tissue, atrophic scars, and telangiectasias, while the included complications comprised ulceration and functional alteration. The authors of the present study considered as recent recurrence those that occurred between 11 months and two years and 11 months of age and as late recurrence those that occurred from three years of age.

The review of medical records of the selected patients was performed, as well as the photographic analysis. Any variable not found in the medical record was investigated during the routine medical consultation or by telephone contact. Patients whose parents did not sign the Free and Informed Consent Form were excluded from the study.

Demographic and prenatal variables of the selected patients were collected at the first consultation in the Dermatology Unit of Universidade Federal de Ciências da Saúde de Porto Alegre. Clinical, therapeutic and treatment response variables were analyzed during routine consultations and through photographic analysis.

Group comparisons were made using Student’s *t* test and Pearson’s chi-square or Wilcoxon-Mann-Whitney and Fisher’s exact tests, when appropriate. The association between the length of treatment with propranolol and clinical variables was tested using the generalized estimating equation (GEE) modeling. For this analysis, a gamma distribution with a logarithmic function was chosen. A robust estimator and an independent method were selected for the covariance and correlation matrices, respectively. The alpha was set at 5%, and the data were analyzed using SPSS 18.0 (SPSS Inc. Chicago, IL, USA). Yates continuity correction was used to evaluate the correlation.

## Results

Between 2006 and 2018, 190 patients (72.6% female) diagnosed with IH were included. The mean age at diagnosis of IH was, in general, 14 days of life, although 63% of the cases had a precursor lesion at birth. The mean age at the diagnosis for superficial IH was 11.9 days, 14 days for mixed IH, and 34 days for deep IH (p = 0.0192).

Focal IH was the most prevalent subtype, occurring in 133 patients (71.8%). Regarding the morphological subtype, most (62.5%) had a mixed component and 74.8% were located on the head/neck. Of the patients with multifocal IH, 25% had visceral hemangioma, with the liver being the most common site (75%; Table 1).

**Table 1:** Infantile hemangiomas: clinical-epidemiological profile and obstetric evolution.

Variables	
Sex – n (%)	190 (100)
Female	138 (72.6)
Male	52 (27.3)
Morphological subtype – n (%)	176 (93)
Superficial	54 (30.6)
Mixed	110 (62.5)
Deep	12 (6.8)
Location – n (%)	187 (98)
Head and neck	140 (74.8)
Anogenital	14 (7.4)
Outro	75 (40.1)
Clinical subtype – n (%)	185 (97)
Focal	133 (71.8)
Segmental	36 (19.4)
Multifocal	16 (8.7)
PHACES syndrome	9 (4.8%)
Delivery – n (%)	127 (67)
C-section	102 (80.3)
Vaginal	25 (19.6)
Family History of IH – n (%)	89 (47)
Yes	19 (21.3)
No	70 (78.6)
Premature birth (<37 weeks) – n (%)	145 (76)
Yes	40 (27.5)
No	105 (72.4)

Thirty-six patients (19.4%) had segmental IH and 4.8% had PHACES syndrome, according to the diagnostic criteria proposed by Metry et al.^12^ One patient had SACRAL syndrome (Table 1).

Forty children (27.5%; 40/145) were preterm (gestational age <37 weeks). The mean birth weight was 2980 g, but 18% had low birth weight (<2500 g) (Table 1).

The mean time of follow-up was 330 days. There was some loss to follow-up (42/190; 22%), and all cases comprised small superficial IH and, therefore, did not require treatment. Twenty-four percent of the patients (46/190) had some type of complication. Of these, ulceration occurred in 29 patients (63%) and functional alteration in 17 patients (36%). Considering all functional alterations, 76.4% occurred in segmental IH and 23.5% in focal IH. The most frequent functional changes were ocular occlusion (9/17: 52.9%) – causing amblyopia or astigmatism – followed by respiratory dysfunction (6/17: 35.2%). All IH that caused respiratory distress were located in the lower lip. On correlation evaluation, ulceration was statistically related to mixed IH (p = 0.004), segmental IH (p < 0.01) and location on the gluteal region (p = 0.001). The presence of superficial IH was related to the absence of ulceration (p = 0.035).

**Figure 1:**
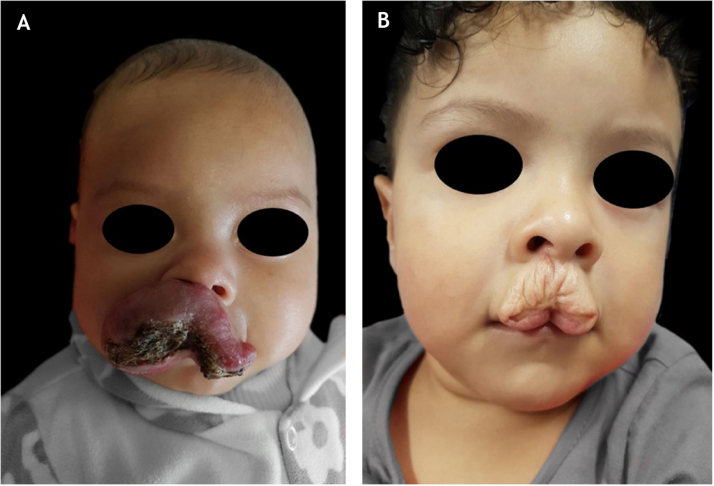
Unaesthetic sequela: (A), patient with ulcerated infantile hemangioma on the lip at 4 months of age and (B), scar and redundant skin at 2 years of age.

Of the treated patients, thirty-one percent (51/163) had some unaesthetic sequelae. Of these, mixed IH was the most common subtype (28/51; 54.9%; p = 0.004), followed by deep IH (12/51; 23.5%; p = 0.8) and superficial IH (11/51; 21.5%; Fig. 1). Fifty-three percent (27/51) of segmental IH (p < 0.01) and 6 of 9 patients with PHACES syndrome had some unaesthetic sequelae (Fig. 1).

Most patients required some treatment (163/190; 86%). Systemic treatment was required in 108 patients (56.8%). Propranolol (2 mg/kg/day) alone was used in 82 cases and propranolol combined with prednisolone (1 mg/kg/day) in 26 cases. Topical 0.5% timolol was used in 55 cases (28.9%). Ninety percent of the patients who received propranolol alone or in combination with steroids had an 80% or greater reduction in hemangioma size at the end of the treatment.

**Figure 2:**
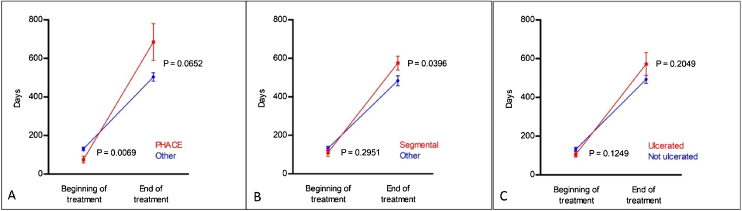
*Post-hoc* comparisons using generalized estimating equation (GEE) modeling related to propranolol treatment time, described in Table 2. (A), PHACES; (B), Segmental hemangiomas and (C), Ulcerated hemangiomas.

Regarding the duration of treatment with propranolol, the mean time of use was 13.15 ± 7.2 months. There was no statistical difference in treatment duration between the different morphological subtypes (Table 2). Regarding the other clinical subtypes, patients with PHACES syndrome required longer treatment (p < 0.001; Table 2); earlier treatment (p = 0.0069) and ended treatment later, but without statistical significance (p = 0.0652; Fig. 2A). Segmental IH also required prolonged treatment, although without statistical significance in the generalized estimating equation (GEE) modeling (p = 0.0407; Table 2); however, with statistical significance in the *post-hoc* analysis both at the start of the treatment (p = 0.029) and at the end (p = 0.0396; Fig. 2B). A significant association was found between ulceration and propranolol treatment duration detected with the GEE modeling (p = 0.0088; Table 2). However, the *post-hoc* analysis found no statistical difference between the groups (ulcerated and non-ulcerated; Fig. 2C).

**Table 2:** Statistical analysis of treatment time with propranolol and multiple variables.

	p-value	n
**Clinical subtype**		
Cyrano	0.8752	120/89
PHACES	<0.0001	120/89^a^
Multifocal IH	0.7953	120/89
Segmental	0.0407	123/89^b^
**Morphological subtype**		
Superficial	0.7747	120/89
Mixed	0.4959	120/89
Deep	0.3167	120/89
**Location**		
Head and neck	0.1196	121/87
Anogenital	0.1361	121/87
Lips	0.4820	123/89
Nose	0.0718	123/89
Ear	0.1231	123/89
Other	0.3215	121/87
**Ulceration**	0.0088	123/89
**Start of Propranolol (before or after 5 months)**	< 0.0001	122/88^c^

Test using the Generalized Estimating Equation (GEE) modeling.

**Figure 3:**
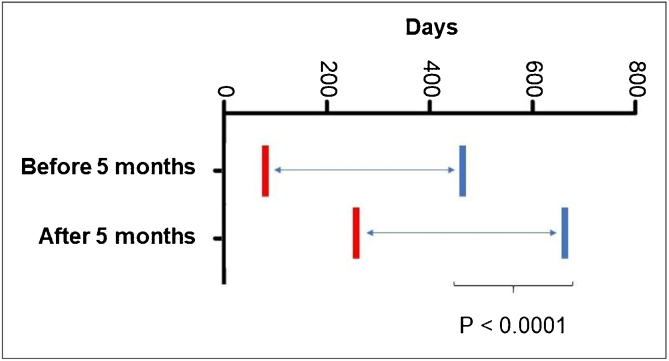
*Post-hoc* comparisons using generalized estimating equation (GEE) modeling described in Table 2, related to the age at the start of treatment with propranolol.

^a,b,c^*Post-hoc* analysis in Graphs 1 A–C (Fig. 2) and Graph 2 (Fig. 3).

As for the age at the start of propranolol treatment, 5.68% started it before 30 days of age; 36.58% after two months; 53.65% after three months; 69.09% after four months and 79.4% after five months of age. It was also observed that patients who started treatment after the age of five months required prolonged treatment time (p < 0001; Fig. 3).

**Figure 4:**
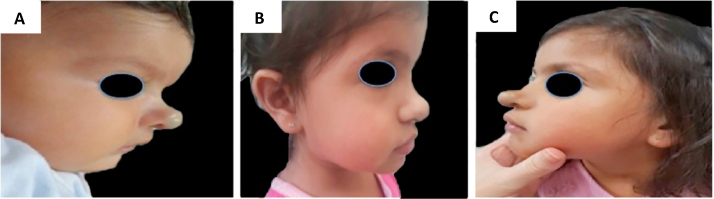
Example of late infantile hemangioma growth 1. (A), Profile of patient with cyrano hemangioma at 2 months of age before treatment. (B), Profile of patient at the end of treatment at 3 years and 2 months of age with propranolol. (C), Profile of the patient at 4 years and 7 months, with late growth.

**Figure 5:**
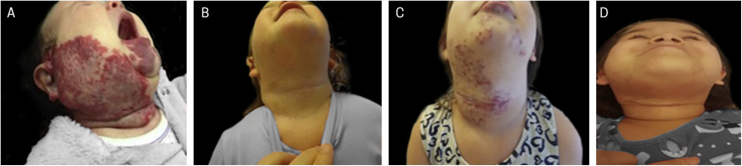
Example of late infantile hemangioma growth. (A), Patient with PHACES syndrome at 2 months of age before treatment. (B), Patient aged 1 year and 10 months – at the end of treatment with propranolol. (C), Patient at 4 years and 6 months of age with late infantile hemangioma growth, when treatment with propranolol was restarted. (D), Patient at 6 years of age, showing regression of the hemangioma.

Recurrences occurred in 16.6% of the treated patients (18/108), and recurrences were not observed in patients using timolol only. All were female (p = 0.005); 11 were mixed IH (11/18; 61.1%; p = 0.5), and 7 were deep IH (7/18; 38.8%; p = 0.37). Early recurrence occurred in eight patients (8/108; 7.4%) and late recurrence in ten patients (10/108; 9.2%). All but one had IH located on the head/neck (17/18; 94.4%; p = 0.012), and ten were segmental IH (10/18; 55.5%; p = 0.007). The IH that most often showed recurrence were those affecting the S3 segment of the face (7/18), upper eyelid (7/18), cyrano subtype (5/18), and those involving the parotid (5/18). Regarding late growth, 8/10 patients had mixed subtypes, 4/10 had PHACES syndrome and 4/10 patients had multiple affected segments (Table 3; Figure 4, Figure 5).

**Table 3:** Recurrent infantile hemangiomas: clinical picture, age at recurrence and treatment.

Patient	Body segment	Subtype	Morphology	Topography	Age at the start and end of treatment	Age at recurrence	Treatment
1	Head and neck	Deep	Focal	Upper lid	Start: 7m	1a 9m	PPL
					End: 1a 6m		
2	Anogenital area	Mixed	Focal	Vulva	Start: 2m	1a 2m	PPL + prednisolone
					End: 1y 1m		
3	Head and neck	Mixed	Segmental	S1, S2, S3, S4, parotid, cyrano, upper lid PHACES	Start: 2m	5a	PPL
					End: 2y		
4	Head and neck	Mixed	Segmental	S2, S3, parotid, lower lip PHACES	Start: 2m	4a	PPL
					End: 2y		
5	Head and neck	Deep	Focal	Upper lid	Start: 5m	1a 7m	PPL
					End: 1y 4m		
6	Head and neck	Deep	Focal	Cyrano	Start: 1m	1a 3m	PPL
					End: 8m		
7	Head and neck	Mixed	Segmental	S1, S2, upper lid	Start: 3m	2a 2m	PPL
					End: 1y 8m		
8	Head and neck	Mixed	Segmental	S1, S2, S3, cervical, parotid, upper lid	Start: 3m	3a	PPL + prednisolone
					End: 1y 7m		
9	Head and neck	Deep	Focal	Cyrano	Start: 2m	4a 7m	PPL
					End: 3y 2m		
10	Head and neck	Deep	Focal	Upper lid	Start: 6m	3a 4m	PPL
					End: 2y 1m		
11	Head and neck	Mixed	Segmental	S3, cervical, lips, PHACES	Start: 3m	3a	PPL + prednisolone
					End: 2y 10m		
12	Head and neck	Deep	Focal	Upper lid	Start: 4m	1a 3m	PPL
					End: 8m		
13	Head and neck	Mixed	Focal	Cyrano	Start: 3m	1a 4m	PPL
					End: 1y		
14	Head and neck	Deep	Segmental	S3, parotid	Start: 6m	2a 6m	PPL
					End: 2y 5m		
15	Head and neck	Mixed	Segmental	S3, parotid, lip, PHACES	Start: 3m	4a	PPL + prednisolone
					End: 1y 3m		
16	Head and neck	Mixed	Segmental	S2, S3, cervical, lips, PHACES	Start: 3m	3a 10m	PPL
					End: 3y		
17	Head and neck	Mixed	Segmental	S4, lips, cyrano	Start: 2m	6a	PPL + prednisolone
					End: 2y 1m		
18	Head and neck	Mixed	Segmental	S2	Start: 8m	3a 5m	PPL
					End: 2y 10m		

S1, Frontotemporal segment; S2, Maxillary segment; S3, Mandibular segment; S4, Frontonasal segment; PPL, Propranolol.

## Discussion

The classification of IH regarding extension, topography, and subtypes can predict the risk of complications, sequelae, and recurrence. Multiple lesions are associated with visceral involvement (especially liver and gastro intestinal tract).^3^, ^9^, ^13^, ^14^, ^15^ Segmental and facial IH show a high risk of unaesthetic sequelae, complications, and recurrence.^9^, ^11^, ^13^ However, there are yet no specific guidelines for the treatment of IH located on this topography, and that have a higher risk of recurrence. It seems obvious to think that for IH at high risk for complications, treatment with propranolol should be prolonged.

It is known that some IH may have a prolonged growth phase, associated with early recurrence, observed after propranolol dose reduction or at the end of the treatment.^16^ On the other hand, to date, there is no scientific explanation for late recurrence after three years of age. The prevalence of late recurrence is scarcely known.^13^, ^17^ Ten to thirty percent of hemangiomas recur after the use of propranolol, and it is estimated that 1% of patients have a late growth phase (after three years of age). Factors associated with recurrence remain uncertain.^13^, ^14^, ^15^, ^16^, ^17^ In this study, the frequency of late growth was high, which makes one think that late recurrence cases may be underestimated. Further studies are required to assess the true prevalence of late recurrence. The most important finding of this study concerns the risk factors for recent and late recurrence. The study results are in agreement with those of the important study by O'Brien et al., except that there are more cases of mixed IH with late growth in the present study.^13^ Interestingly, it seems that recent and late recurrences share the same risk factors, so these patients should be monitored throughout childhood. In this study, patients with segmental IH and PHACES syndrome required prolonged treatment. This finding was expected because these patients constitute more severe cases and have more recurrences, which resulted in prolonged treatment time.

In a previous cohort including 1,096 children, Metry et al. found 20% of segmental IH and 2.3% of PHACES syndrome in the total number of cases.^10^ The present study found a higher frequency of PHACES syndrome and segmental IH. A possible reason for this finding is that the present study was carried out in a tertiary center, a referral center for severe cases.

Ulceration is estimated to occur in approximately 5% to 21% of IH and was the most common complication in a prospective study, occurring in 16% of the cases.^18^, ^19^, ^20^, ^21^ Ulceration can lead to severe pain, bleeding, and secondary infection, in addition to the risk of permanent scarring.^19^

Early intervention and/or referral to a specialized service as of the fourth week of life are recommended in cases of IH with a high risk for complications (ulceration, unaesthetic sequelae or functional loss), aiming to reduce the risk of unaesthetic scarring and permanent sequelae.^22^ In the present study, only 50% of the patients started treatment before the age of three months and this fact may have contributed to unaesthetic sequelae. It is noteworthy the high frequency of sequelae in the present study. Difficulty in access to tertiary care and/or lack of information by pediatricians and dermatologists can explain this delay. The present study showed that children who started treatment before four months of age had a better response to treatment and children who started treatment at an older age required prolonged treatment.

The limitations of the present study were the lack of data and photographs in some medical records and loss to follow-up, as this was a retrospective study, and because it was carried out in a single hospital, it does not constitutes a population-based study.

## Conclusion

This is the first study to draw a broad epidemiological profile of IH in southern Brazil. Mixed, segmental IH and those located in the gluteal region are at risk for ulceration. Mixed, segmental IH and those localized in the head and neck are at risk for sequelae. Female sex and segmental IH are risk factors for recurrence. Additionally, IH located on the S3 segment of the face, upper eyelid, lips, affecting the parotids and cyrano subtype add greater risk for complications. The present study establishes the risk factors for recurrence and alerts to the fact that these patients must be carefully monitored even after treatment interruption. Specific treatment protocols are required for IH with a high risk for complications and recurrence.

## Financial support

The study was funded in part by the 10.13039/501100002322Coordination for the Improvement of Higher Education Personnel – Brazil (*Coordenação de Aperfeiçoamento de Pessoal de Nível Superior* – CAPES) – Funding Code 001.

## Authors’ contributions

Letícia Gaertner Mariani: Design and planning of the study; collection of data; drafting of the manuscript.

Lílian Moraes Ferreira: Design and planning of the study; collection of data.

Diego Luiz Rovaris: Analysis and interpretation of data.

Renan Rangel Bonamigo: Analysis and interpretation of data.

Ana Elisa Kiszewski: Design and planning of the study; analysis and interpretation of data; critical review of the manuscript.

## Conflicts of interest

Ana Elisa Kiszewski is a consultant for Johnson & Johnson. The other authors declare no conflicts of interest.
